# The Integrated Management of Sugar-Beet Soilborne Diseases Through Rhizosphere Microbiome Strategies: A Critical Review of Agronomic Evidence and Application Gaps

**DOI:** 10.3390/plants15182798

**Published:** 2026-09-12

**Authors:** Yue Chang, Wenjin Chen, Liang Wang, Ziqiang Zhang

**Affiliations:** Inner Mongolia Academy of Agricultural and Animal Husbandry Sciences, Hohhot 010031, China

**Keywords:** sugar beet, *Beta vulgaris*, soilborne disease, rhizosphere microbiome, biological control, synthetic microbial community, disease-suppressive soil, integrated pest management

## Abstract

Soilborne diseases caused by *Rhizoctonia solani*, *Fusarium oxysporum* f. sp. *betae*, *Aphanomyces cochlioides*, and *Pythium* spp. constrain sugar-beet production worldwide, with yield losses exceeding 50% in severely affected fields. These pathogens frequently co-occur, yet most biological control and microbiome studies address them individually, and recommendations from model crops often fail to translate to sugar beet’s distinctive root biology and rotation systems. This critical review synthesises evidence on the rhizosphere microbiome as a plant protection resource, organising the literature by agronomic applicability: crop rotation, organic amendments, soil physicochemical management, microbial inoculants and synthetic communities, and microbiome-informed breeding and diagnostics. For each strategy, we state the evidence directness (direct sugar-beet field trials, greenhouse data, cross-crop extrapolation, or mechanistic inference) and evaluate the gap between experimental efficacy and field-ready recommendations. Direct field evidence remains limited: the best-characterised example is *Rhizoctonia*-suppressive soil linked to non-ribosomal peptide synthetase (NRPS)-producing Pseudomonadaceae and Burkholderiaceae, with 2,4-DAPG-producing *Pseudomonas* populations also being correlated with disease suppression in sugar-beet seedlings, plus one integrated fungicide-biocontrol field trial. We identify multi-pathogen challenge experiments, multi-site field validation of synthetic communities, and microbiome-informed breeding as priority research gaps. Rhizosphere microbiome management should be integrated into existing disease programmes rather than deployed as a stand-alone replacement.

## 1. Introduction

Sugar beet (*Beta vulgaris* L.) is the world’s second-largest sugar crop, accounting for approximately 20% of global sugar production and underpinning the agricultural economies of Europe, North America, and northern China [1,2]. The crop also serves as feedstock for bioethanol production, animal feed, and functional food ingredients, giving it broad economic significance [3]. Yet sugar-beet production has long been constrained by soilborne diseases. Yield losses from individual soilborne pathogens can exceed 50% annually, with total crop failure reported in severely affected fields [4]. In China, the concentration of sugar-beet cultivation in regions such as Inner Mongolia and Xinjiang [1,2], coupled with extended continuous cropping, has intensified soilborne disease pressure to the point where it now constitutes the primary biotic stress limiting sustainable development of the industry.

Growers have long managed these diseases through three measures—chemical fungicides, resistant cultivars, and rotation design—and each is now strained. Fungicide use carries documented costs in environmental contamination, disruption of non-target microbes, and selection for resistant pathogen populations [5]. On the genetic side, resistant germplasm is scarce; the *Rhizoctonia*-resistant sugar-beet cultivars reported to date tend toward partial resistance, which breaks down under high inoculum loads [6]. Rotation, the third lever, is undermined when economic pressure shortens the cycle, and *Rhizoctonia* sclerotia persist in soil for several years, eroding the benefit of even a well-planned sequence [4,7]. The policy environment has tightened in parallel: the EU Green Deal including its Farm-to-Fork strategy targeting a 50% reduction in pesticide use by 2030, and China’s Pesticide Reduction Initiative both push toward greener, more durable disease control [8].

The rhizosphere microbiome sits inside the plant holobiont framework and offers a route to disease suppression that is real but uneven across contexts [9,10]. Disease-suppressive soils were noticed as far back as the late nineteenth century, and their microbial basis has been unpacked in stages. Baker and Cook [11] gave the phenomenon its first systematic treatment. Mechanistic work followed: Mendes et al. [12], working with sugar-beet soils, used PhyloChip-based community profiling coupled with culture-dependent analysis to tie *Rhizoctonia* suppression to Gammaproteobacteria governed by non-ribosomal peptide synthetase gene clusters, while other groups framed the rhizosphere as a contest between pathogens and beneficial microbes [13,14]. Schlatter et al. [15] later pulled suppressive-soil research into mainstream microbiome ecology, arguing that network complexity and functional redundancy—rather than any single taxon—stabilise suppressiveness. The question that matters for agronomists is narrower than “can the microbiome suppress pathogens?” Under controlled conditions it plainly can. The question is whether that capacity can be engineered or reinforced through practices a grower can actually deploy in the field. This review weighs the evidence for each major strategy against that standard.

Four features of sugar beet make it hard to directly extrapolate microbiome recommendations from other crops. Its roots release organic acids—including citramalic and salicylic acid, whose exudation is enhanced under P deficiency, and which contribute to soil phosphorus mobilisation [16], thereby creating a distinctive rhizosphere chemical environment. Sugar beet also accumulates glycine betaine as an important tissue osmolyte; however, direct evidence that glycine betaine is released through the roots and subsequently contributes to microbial recruitment or microbiome assembly in *Beta vulgaris* remains limited. More broadly, sugar-beet root exudates have been shown to influence rhizosphere microbial diversity and community composition [17], and this exudate landscape is increasingly read as a dynamic rather than static filter. The fleshy taproot presents a colonisation surface unlike the fibrous-root systems of most model crops, which changes how biocontrol agents must establish. Sugar beet is also generally non-mycorrhizal [18], although mycelium from neighbouring mycorrhizal hosts can modulate its defence responses; suppressive functions still rely largely on free-living rhizosphere bacteria and fungi. Finally, the three-year rotation with its postharvest bare-soil phase imposes a distinctive successional rhythm on the microbial community. Taken together, results from wheat, tomato, or *Arabidopsis* cannot be carried over to sugar beet without local field confirmation.

**Figure 1:**
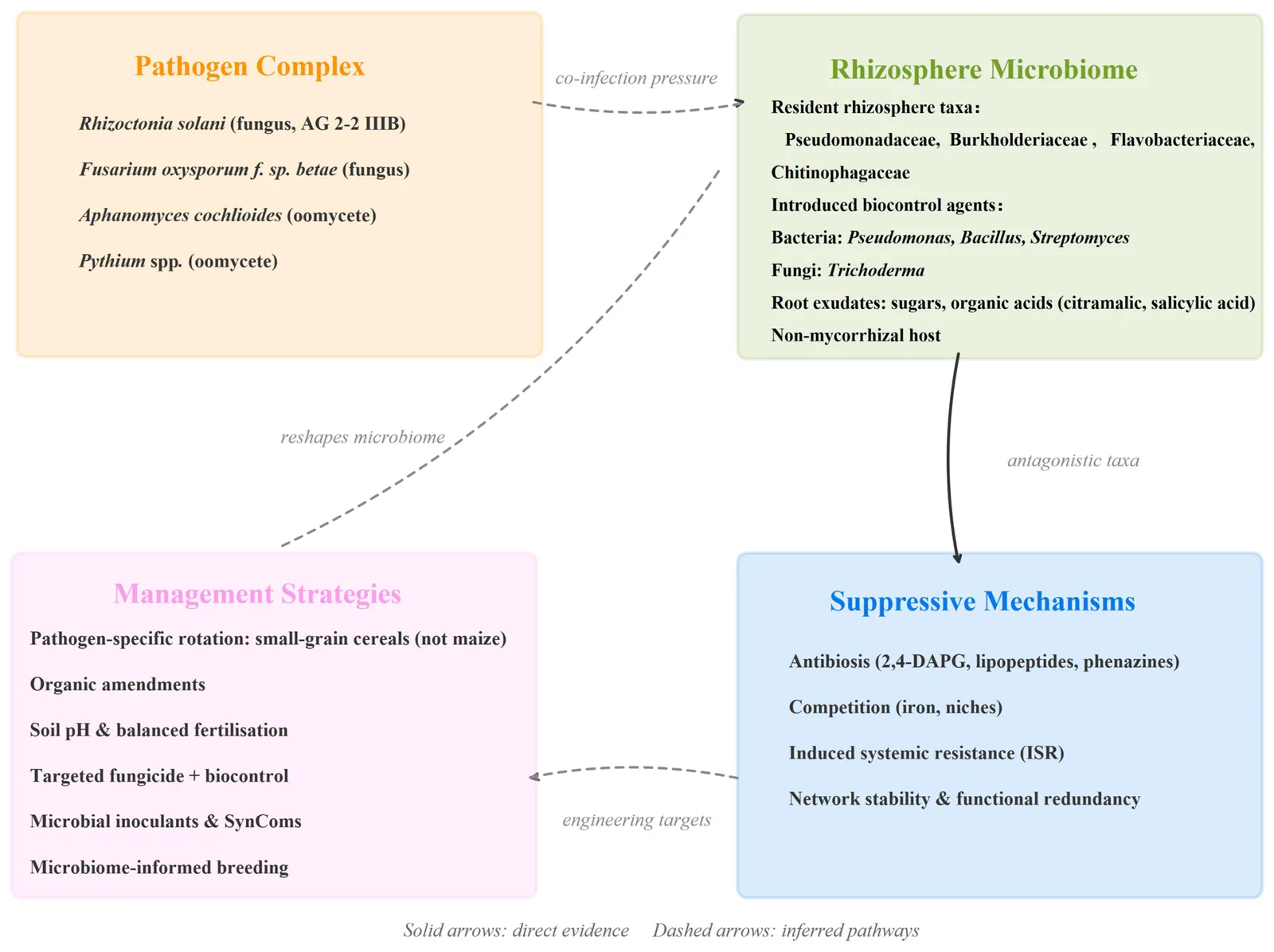
Conceptual framework for integrated management of sugar-beet soilborne diseases through rhizosphere microbiome strategies. Solid arrows indicate pathways supported by direct sugar-beet field or greenhouse evidence; dashed arrows indicate pathways inferred from other crops or mechanistic reasoning. The framework depicts interactions among the pathogen complex (*Rhizoctonia solani*, *Fusarium oxysporum* f. sp. *betae*, *Aphanomyces cochlioides*, and *Pythium* spp.), the rhizosphere microbiome (shaped by root exudates and core microbial taxa), suppressive mechanisms (antibiosis, competition, induced systemic resistance, and network stability), and management strategies (crop rotation, organic amendments, soil physicochemical management, biocontrol agents, synthetic communities, and microbiome-informed breeding). A feedback loop indicates that management reshapes the rhizosphere microbiome, which in turn modifies pathogen pressure and suppressiveness over time.

This is a critical narrative review, not a systematic review. Several recent reviews cover overlapping ground, including reviews of types of fungal root rot of sugar beet and their management [19], *Rhizoctonia* root-rot diseases in sugar beet [20], and research progress on sugar-beet root-rot control. The present review’s distinct contribution is its evidence-directness grading framework and its explicit separation of pathogen co-occurrence from experimentally demonstrated synergistic co-infection, which together organise the literature by agronomic applicability rather than by microbiological mechanisms. Its aim is to judge how rhizosphere microbiome knowledge can be turned into integrated plant protection for sugar beet, with attention to field applicability, fit with existing controls, and the gaps that block routine use. The structure follows management strategies rather than microbiome mechanisms. Section 3 lays out the pathogen complex that any strategy must suppress. Section 4 pulls together the microbiology of disease-suppressive soils as the conceptual base. Section 5, Section 6, Section 7 and Section 8 weigh the main agronomic and microbial strategies, ordered by how direct their evidence is. Section 9 covers emerging diagnostics and breeding tools, and Section 10 sets out priority research gaps. Organising around management actions is meant to make the review useful to researchers, advisers, and crop managers in sugar-beet systems. Figure 1 sketches the conceptual framework: how the pathogen complex, rhizosphere microbiome, suppressive mechanisms, and management strategies interact to set field-level disease outcomes.

## 2. Literature Search and Evidence Appraisal

This review was conducted as a critical narrative review following a transparent literature identification and synthesis approach rather than a systematic review framework. Accordingly, it does not claim adherence to systematic review protocols such as PRISMA. The relevant literature was retrieved from Web of Science, Scopus, and PubMed in March 2026 using combinations of keywords including “sugar beet”, “*Beta vulgaris*”, “rhizosphere microbiome”, “soilborne disease”, “*Rhizoctonia solani*”, “*Fusarium oxysporum*”, *“Aphanomyces cochlioides*”, “*Pythium*”, “disease-suppressive soil”, “biological control”, “*Pseudomonas*”, “*Bacillus*”, “*Trichoderma*”, “*Streptomyces*”, “crop rotation”, “organic amendment”, “synthetic microbial community”, and “microbiome-informed breeding”. The search covered the literature published from January 1970 – March 2026, with no language restriction applied at the search stage but screening limited to English-language and Chinese-language publications. Inclusion criteria were: (i) original research or authoritative reviews in peer-reviewed journals; (ii) studies on sugar beet or closely related crops in the family Amaranthaceae (subfamily Chenopodioideae, formerly treated as the separate family Chenopodiaceae), principally spinach (*Spinacia oleracea*), Swiss chard, fodder beet, and other cultivated *Beta* species [21]—chosen because they share the *Beta*/Chenopodioideae root biology most relevant to rhizosphere microbiome extrapolation; (iii) studies addressing the four target pathogens or their biocontrol agents; and (iv) studies offering mechanistic or field-scale evidence. Exclusion criteria were: studies solely on foliar diseases; studies on pathogens outside the defined complex without cross-crop relevance; and non-peer-reviewed sources, except for regulatory documents. Additional relevant studies were identified through backward citation tracing of key publications and related reviews. The principal Boolean search strings used for Web of Science, Scopus, and PubMed are provided in Table S3. For the preparation and interpretation of research findings, all AI-assisted outputs were critically evaluated and independently verified by the authors, who remain fully responsible for the accuracy, integrity, and scientific validity of the published work.

We prioritised four kinds of sources: original research and authoritative reviews in peer-reviewed journals; work on sugar beet or on closely related crops in the family Amaranthaceae (subfamily Chenopodioideae); studies of the four target pathogens or their biocontrol agents; and studies offering mechanistic or field-scale evidence. To appraise what we found, we used a formal four-tier hierarchy of evidence directness: Tier 1: direct sugar-beet field evidence (randomised or replicated field trials, or multi-site field observations with disease or agronomic endpoints, in the target crop; highest directness and translational relevance); Tier 2: controlled-environment sugar-beet evidence (greenhouse or laboratory studies using sugar beet); Tier 3: cross-crop or otherwise indirect evidence (extrapolated from other crops, including sugar-beet observational or postharvest evidence that does not directly test field soilborne-disease suppression); and Tier 4: mechanistic inference without crop-specific validation (in vitro or purely mechanistic studies; lowest directness and translational relevance). These tiers reflect the directness of evidence and its translational proximity to field conditions, not methodological quality or risk of bias; a rigorously executed controlled experiment (Tier 2) may be methodologically superior to a weakly replicated single-season field trial (Tier 1).

**Figure 2:**
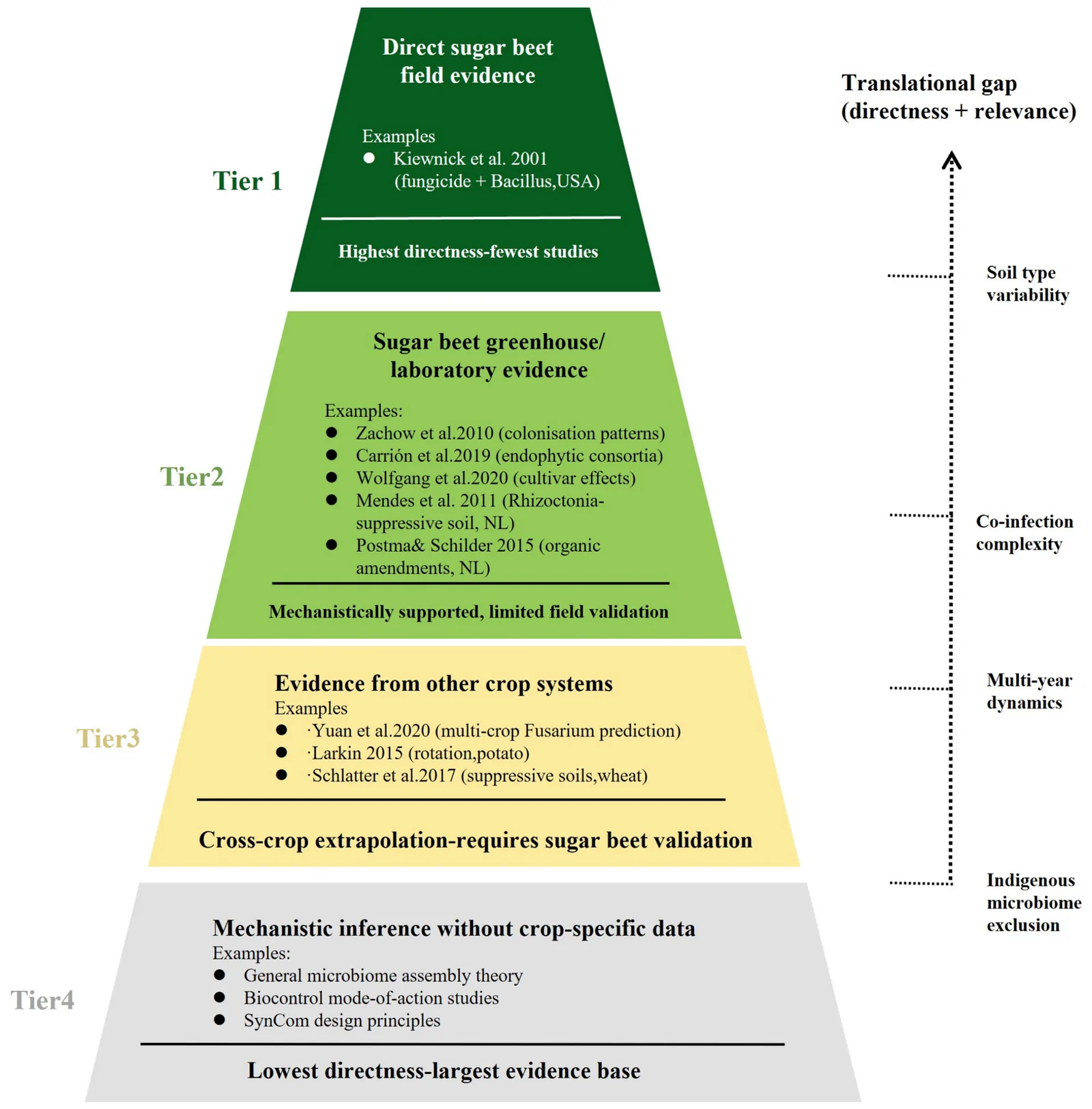
Evidence hierarchy for rhizosphere microbiome strategies in sugar beet and the translational gap to field-ready recommendations. The pyramid shows four tiers of evidence directness: direct sugar-beet field evidence (top, fewest studies), sugar-beet greenhouse/laboratory evidence, cross-crop or otherwise indirect evidence, and mechanistic inference without crop-specific data (bottom, largest evidence base). The right-side arrow annotates the key factors driving the translational gap between laboratory efficacy and field reliability: soil-type variability, co-infection complexity, multi-year dynamics, and indigenous microbiome exclusion. Representative studies shown in the pyramid are Mendes et al. [12], Schlatter et al. [15], Kiewnick et al. [22], Zachow et al. [23], Carrión et al. [24], Yuan et al. [25], Larkin [26], Postma and Schilder [27], and Wolfgang et al. [28]. Tiers rank evidence directness and translational relevance, not study quality.

Each empirical or mechanistic substantive claim in Section 5, Section 6, Section 7, Section 8 and Section 9 is assigned an evidence tier; purely regulatory descriptions and synthesis/recommendation statements are treated as contextual rather than as efficacy evidence. Evidence tiers are assigned to individual claims, not to publications; a single study may contribute evidence at several tiers (e.g., field observations at Tier 1 alongside mechanistic data at Tier 4), and Table S1 notes where a study spans more than one tier. Where consecutive claims share a tier, the tier is stated once and applies until a new tier is indicated. Where a claim rests on correlation rather than causation, or on cross-crop extrapolation rather than sugar-beet data, we say so in the text. This grading matters for agronomic recommendations because the distance between controlled-experiment efficacy and field reliability is the central translational problem. Figure 2 maps the evidence hierarchy for rhizosphere microbiome strategies in sugar beet and the translational gap to field-ready recommendations. Table S1 lists the key studies and records the claim-level evidence tier(s), together with field-design context where applicable. Tier 1 itself spans a range from single-season, single-site trials to multi-year, multi-site programmes; Table S1 records the number of seasons, locations, and field designs for each field study so that Tier 1 evidence is not treated as uniform.

## 3. The Sugar-Beet Soilborne Pathogen Complex as a Management Target

**Figure 3:**
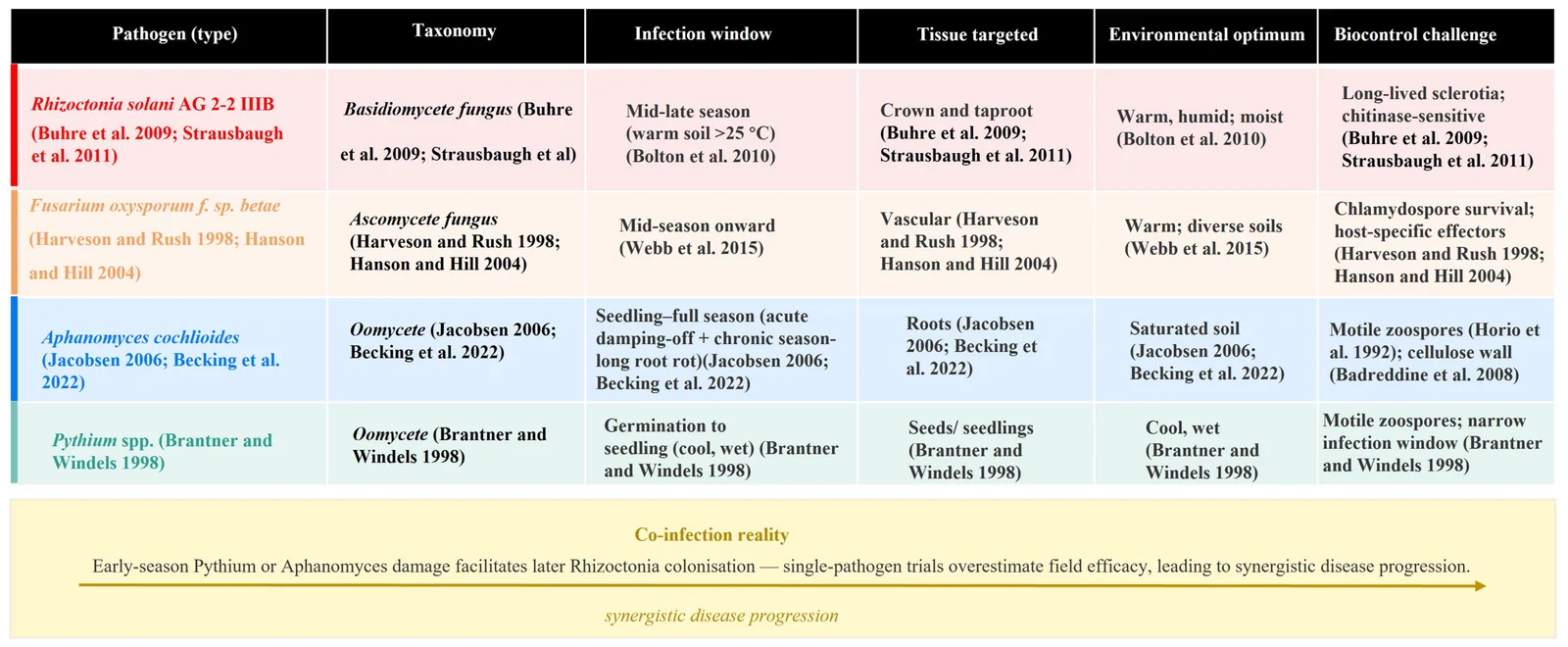
Comparative infection biology of the four principal sugar-beet soilborne pathogens and implications for biocontrol design. The four pathogens differ in taxonomic identity (fungi vs. oomycetes), infection window (seedling vs. mid–late season), targeted tissue (seeds, vascular tissue, crown and taproot), environmental optima, and key biocontrol challenges. The bottom bar highlights the co-infection reality: early-season *Pythium* or *Aphanomyces* damage can facilitate later *Rhizoctonia* colonisation, meaning that single-pathogen experimental designs often overestimate field efficacy. Sources for the pathogen characteristics shown: Buhre et al. [29], Strausbaugh et al. [30], Bolton et al. [31], Harveson and Rush [32], Hanson and Hill [33], Webb et al. [34], Jacobsen [35], Becking et al. [36], Horio et al. [37], Badreddine et al. [38], and Brantner and Windels [39].

To know what agronomic management has to suppress, one first has to characterise the pathogen complex behind sugar-beet soilborne disease. Sugar-beet root and crown rot are not a single-pathogen problem like most model systems; they involve several fungi and oomycetes with different types of infection biology, host ranges, and environmental optima. This complexity shapes management directly. This review focuses on the four principal fungal and oomycete root pathogens—*R. solani*, *F. oxysporum* f. sp. *betae*, *A. cochlioides*, and *Pythium* spp.—that cause the most economically significant soilborne losses. Other soilborne threats, including cyst nematode (*Heterodera schachtii*), rhizomania (Beet necrotic yellow vein virus transmitted by *Polymyxa betae*), and *Sclerotium rolfsii*, are excluded from the primary scope because their management relies on distinct control paradigms (resistant varieties and vector control for rhizomania and nematicides for cyst nematode) that fall outside the rhizosphere microbiome strategies reviewed here, though their interactions with the focal pathogens warrant future investigation. A strategy that works against one pathogen can be useless—or even harmful—against another, and because co-infection is the field norm, single-pathogen experiments tend to overstate what a treatment will achieve in practice. Figure 3 compares the infection biology of these pathogens and spells out what it means for biocontrol design.

### 3.1. Principal Pathogens and Their Infection Biology

Crown and root rot, caused by *Rhizoctonia solani* Kühn (primarily anastomosis group AG 2-2, especially subgroup AG 2-2 IIIB, which is more prevalent in warmer regions and includes isolates pathogenic to maize, and to a lesser extent AG 2-2 IV, which is more common in cooler regions and is generally less aggressive on maize; AG 4 also contributes to seedling damping-off), is among the most economically damaging soilborne diseases of sugar beet across major production regions [29,30]. *R. solani* overwinters in soil as long-lived sclerotia, which can survive for multiple years and substantially compromise the effectiveness of rotation strategies. Disease development is generally favoured by warm and humid conditions [31].

*Fusarium* spp. cause a spectrum of soilborne diseases in sugar beet, including *Fusarium* yellows and *Fusarium* root rot [32,33]. The pathogen colonises vascular tissues, causing yellowing of foliage, wilting, and taproot discolouration, and can survive in soil as chlamydospores for extended periods, with root-rot severity varying significantly among sugar-beet genotypes [40]. Its management is complicated by the genetic diversity of populations and the emergence of new virulent lineages [41], with temperature strongly modulating disease development [34] and recent phylogenetic work revealing that some isolates previously identified as *F. oxysporum* f. sp. *betae* actually belong to *F. secorum* [42,43].

Root rot, caused by the oomycete *Aphanomyces cochlioides*, is important in humid production regions of Europe and North America, with persistent losses reported in the Red River Valley of Minnesota and North Dakota [35,36]. *A. cochlioides* causes both an acute seedling damping-off phase and a chronic season-long root-rot phase that seed treatment does not prevent. Unlike fungal pathogens, *A. cochlioides* produces motile zoospores that actively swim towards host roots in response to chemotactic signals, including cochliophilin A exuded by sugar-beet and spinach roots [37]. This zoospore-mediated infection biology is fundamentally different from hyphal colonisation and has implications for biocontrol: antagonistic mechanisms effective against fungi (e.g., chitinases) have reduced efficacy against oomycete cell walls, which are composed primarily of cellulose and beta-glucans but contain approximately 10% N-acetylglucosamine as non-crystalline chitosaccharides linked to glucans [38] (note: this study concerns *A. euteiches*, and the cross-species extrapolation to *A. cochlioides* is graded Tier 3).

Damping-off caused by *Pythium* spp., particularly *P. ultimum* and *P. aphanidermatum*, is important during seed germination and early seedling establishment under high soil moisture. Their temperature niches differ: *P. ultimum* is generally favoured by cooler to moderate temperatures, whereas *P. aphanidermatum* is a high-temperature pathogen [39]. Like *Aphanomyces*, *Pythium* is an oomycete pathogen associated with damping-off and early seedling diseases in sugar beet. Because infections occur mainly during early plant establishment, disease pressure is concentrated shortly after sowing, when the rhizosphere microbiome is undergoing dynamic assembly through interactions among seed-associated and soil-derived microorganisms [9,13,39].

### 3.2. Pathogen Co-Occurrence and Implications for Management Design

Under field conditions, these pathogens frequently co-occur in the same field. Co-occurrence of *R. solani*, *F. oxysporum*, and *A. cochlioides* in the same field is common [35,36,44]. It is important to distinguish pathogen co-occurrence (presence of multiple pathogens in the same field) from experimentally proven synergistic co-infection (where concurrent infection results in disease severity greater than the sum of individual effects). Buhre et al. [29] reported that *Rhizoctonia* crown-rot severity in German sugar-beet fields was significantly influenced by cultivar susceptibility and crop rotation, with increasing maize proportion exacerbating disease, and Jacobsen [35] noted that *A. cochlioides* and *Pythium* spp. are among the soilborne pathogens that co-occur in the sugar-beet root-rot complex. Proposed mechanisms include early-season infection by *Pythium* or *Aphanomyces* disrupting root tissue integrity and providing entry pathways for subsequent colonisers such as *R. solani*, additive effects of cell wall-degrading enzymes from different pathogens, and depletion of host immune resources by simultaneous activation of defence responses against phylogenetically diverse attackers [44].

This co-infection reality has two direct agronomic consequences. First, effective intervention strategies may need to target both fungal and oomycete pathogens simultaneously, supporting the shift from single antagonistic strains to functional microbial consortia with complementary modes of action [45,46]. Second, the predominant use of single-pathogen inoculation designs in published sugar-beet biological control studies—for example, Kiewnick et al. [22] tested a *Bacillus* isolate only against *R. solani*, and Zachow et al. [23] evaluated antagonist colonisation solely in the context of *Rhizoctonia*—creates a methodological disconnect from field co-infection reality [23]. Agronomic recommendations based on single-pathogen trials should therefore be interpreted with caution, because single-pathogen efficacy may not predict performance under co-infection, where pathogen interactions can be antagonistic, synergistic, or neutral.

## 4. Microbiological Basis of Disease-Suppressive Soils: The Conceptual Foundation for Management

Disease-suppressive soils provide the conceptual foundation for microbiome-based management. Baker and Cook [11] first defined suppressive soil as soil in which a pathogen does not establish or persist, establishes but causes little or no disease, or establishes and causes disease at first but then the disease declines with continued monoculture. Weller et al. [47] classified suppressiveness into two types: general suppressiveness, derived from the collective metabolic activity of the total soil microbial biomass and not transferable between soils, and specific suppressiveness, mediated by particular microbial taxa or functional groups and transferable via small amounts of soil inoculum. General suppressiveness is enhanced by any management practice that increases microbial biomass and activity, such as organic matter addition, whereas specific suppressiveness depends on the enrichment of key antagonistic populations through repeated pathogen pressure, with plants actively shaping their rhizosphere microbiome through root exudate composition to create disease-suppressive soil legacies [48]. Schlatter et al. [15] proposed that these two forms are endpoints of a continuum, with microbial network complexity and functional redundancy determining the type and stability of suppressiveness. For sugar beet, general suppressiveness may be enhanced through organic amendments and diversified rotations (Section 5 and Section 6), whereas specific suppression of *Rhizoctonia solani* has been associated with the enrichment and activation of particular bacterial taxa and antifungal functions in the rhizosphere and root endosphere [12,24].

Disease decline—the phenomenon in which disease severity naturally subsides after an initial peak during continuous monoculture—represents one of the most compelling lines of evidence for microbially mediated suppressiveness. The best-characterised example is wheat take-all decline, where continuous wheat cultivation for four to six years leads to the build-up of 2,4-DAPG-producing *Pseudomonas* populations that suppress *Gaeumannomyces tritici* [15,47,49]. 2,4-DAPG is an important antifungal determinant contributing to root defence in several plant-beneficial *Pseudomonas* strains [49,50]. In sugar beet, disease decline has been anecdotally reported in *Rhizoctonia* systems, but the peer-reviewed evidence base is far less solid than for wheat, even though take-all suppressive soils show distinct microbial community patterns relevant to understanding such phenomena [51]. The practical relevance of disease decline is also constrained by the fact that sugar beet is rarely grown in continuous monoculture for more than two to three years, making it uncertain whether the four-to-six-year decline trajectory observed in wheat can be realised in commercial sugar-beet production. Moreover, under continuous monoculture, pathogen accumulation typically outweighs any beneficial microbial enrichment—which is precisely why crop rotations were developed as the primary agronomic countermeasure.

Several candidate microbial markers for suppressiveness have emerged from culture-independent studies. Mendes et al. [12], working specifically in sugar-beet soils, identified Gammaproteobacteria (particularly Pseudomonadaceae) and Betaproteobacteria (Burkholderiaceae) as enriched in *Rhizoctonia*-suppressive soils and linked suppressiveness to non-ribosomal peptide synthetase (NRPS) gene clusters (enrichment of these taxa is associative; causal suppressive function is supported only where the metabolite has been functionally validated, such as for thanamycin [52]). The chlorinated lipopeptide thanamycin, produced by *Pseudomonas* sp. SH-C52 isolated from the rhizosphere of sugar-beet grown in this suppressive soil, has been identified as an important determinant of the strain’s antifungal activity against *R. solani* [52]. Carrión et al. [24] identified Flavobacteriaceae and Chitinophagaceae as markers of *Rhizoctonia* suppressiveness in sugar-beet soils. Yuan et al. [25], in a multi-crop study using global metadata, identified Sphingomonadaceae, Xanthomonadaceae, *Streptomyces*, and Bradyrhizobiaceae as key bacterial predictor taxa (predictive associations from cross-crop metadata, not functionally confirmed in sugar beet) and *F. oxysporum* and *Mortierella* as key fungal predictors of disease status. At the functional level, genes encoding 2,4-DAPG biosynthesis (phlD)—whose abundance correlates with *R. solani* suppression in sugar-beet seedlings [53]—chitinases, and siderophore production have been proposed as functional markers that may prove more reliable than taxonomic indicators [45]. Three challenges, though, limit the current agronomic utility of microbial markers: taxonomic markers may mask substantial functional heterogeneity within a family; markers identified in one soil type or climate zone often fail to generalise; and quantitative detection methods for functional genes in field soils are not yet standardised for routine diagnostic use. Marker-based diagnostics are therefore a promising but not yet field-ready tool (Section 9). A further limitation is the restricted reproducibility of suppressiveness transfer: enriching or transplanting suppressive microbial communities into non-suppressive soils frequently fails, and the effect is strongly dependent on local soil type, climate, and indigenous microbiota. This context-dependence limits the agronomic utility of suppressive-soil markers identified in one location for deployment in another.

## 5. Crop Rotation and Continuous Cropping Management

Rotation with well-chosen non-host or low-susceptibility crops is the agronomic lever most growers can pull first to steer the rhizosphere microbiome against soilborne disease. A good rotation cuts *R. solani* and *F. oxysporum* inoculum density while reshaping the microbial community and raising the abundance of disease-suppressive functional groups and antagonists [26,54], with crop rotational diversity demonstrably increasing the disease-suppressive capacity of soil microbiomes (Tier 3, cross-crop) [54] and reintroducing native bacterial strains lost under monocropping restoring disease suppression in a peanut system [55]. Two mechanisms are at work. Non-host rotation crops starve pathogens of susceptible host tissue and break their life cycle, and they sustain a more diverse microbial community that lifts general suppressiveness. In sugar beet specifically, Zachow et al. [56] surveyed the indigenous antagonistic potential of sugar-beet-associated microbial communities across microhabitats and developmental stages (a culture-dependent antagonist survey, not a rotation experiment) (Tier 3, indirect sugar-beet field survey; no disease-suppression endpoint) and found that this potential depended heavily on the target pathogen, the location, and the plant’s growth stage. Table S2 summarises current microbiome-oriented management practices for sugar-beet soilborne disease control and their main limitations. Rotation crop choice must be pathogen-specific: small-grain cereals (wheat and barley) are low-susceptibility hosts for *R. solani* AG 2-2 IIIB and are preferable rotation partners, whereas maize (*Zea mays*) is a confirmed host of AG 2-2 IIIB [29,57] and should be avoided or limited in rotations targeting *Rhizoctonia* suppression. Bean crops are the most susceptible, corn is intermediate, and wheat is the least susceptible (Tier 1, sugar-beet field) [57]. Tillage is a further first-order determinant of both *Rhizoctonia* inoculum dynamics and microbial community structure: minimum tillage can conserve suppressive microbial communities but may also concentrate inoculum near the surface, whereas deeper tillage can bury sclerotia but disrupts established rhizosphere communities. Buhre et al. [29] reported that *Rhizoctonia* crown-rot severity was significantly influenced by cultivar susceptibility and crop rotation, with tillage interacting with rotation effects. Cover crops and green manure are standard in beet rotations and connect directly to the biofumigation discussion in Section 6: cruciferous cover crops release isothiocyanates with biofumigant activity, but their susceptibility to certain sugar-beet pathogens [4] must be weighed against their suppressive potential.

Rotation crop choice is critical, though, and not universally beneficial. Crucifers may share pathogens with sugar beet (Tier 1, sugar-beet field) [4], and certain rotation sequences can inadvertently build inoculum of pathogens with broad host ranges. The optimal rotation duration and crop sequence for microbiome-mediated disease suppression in sugar beet have not been established through controlled field trials; recommendations should therefore be based on local trial data rather than universal prescriptions, as continuous cropping of sugar beet reshapes rhizosphere microbial communities and enriches pathogenic taxa over time (Tier 1–2, sugar-beet field) [58,59]. Continuous cropping obstacles involve not just pathogen accumulation but also beneficial microorganism diversity decline, allelopathic inhibition, and autotoxin accumulation (Tier 3, cross-crop) [60,61], with recent studies showing that continuous sugar-beet cropping alters soil microbial diversity, with bacterial diversity generally declining, while fungal responses are compartment- and duration-dependent [58,59] and trigger substantial transcriptomic and metabolomic changes in sugar-beet roots (Tier 2, sugar-beet controlled) [62]. Direct field evidence reinforces this link: Kusstatscher et al. [63] found that disease incidence across sugar-beet fields correlated with microbial diversity and distinct biological markers, supporting the premise that diversity loss under continuous cropping is itself a disease-risk signal (Tier 1, multi-site field, correlative). In Chinese production regions where shortened rotations are economically driven, the trade-off between short-term profitability and long-term inoculum build-up is a central management tension that a microbiome-aware rotation design could help resolve, but only if backed by region-specific field data.

A key evidence gap is the lack of quantitative, multi-site sugar-beet rotation trials that simultaneously measure pathogen inoculum dynamics, microbial community composition, and disease incidence over multiple rotation cycles. Most rotation recommendations for sugar beet rest on extrapolation from other crops or on observational field data without microbiome monitoring, though long-term-cropping-system studies on potato demonstrate that disease-suppressive rotations alter soil microbial communities cumulatively over time (Tier 3, cross-crop). This gap is particularly consequential for Inner Mongolia and Xinjiang, where continuous cropping pressure is most intense, and where local soil and climate conditions may modify rotation effects observed elsewhere.

## 6. Organic Amendments and Biofumigation

Organic amendments can lift soil disease suppressiveness by raising microbial activity and biomass and by shifting community composition, though effects on diversity depend on the amendment and the soil (Tier 3, cross-crop) [64,65,66,67], and bio-organic fertilizers in particular stimulate indigenous *Pseudomonas* populations that strengthen disease suppression (Tier 3, cross-crop) [68]. One of the clearest direct sugar-beet-specific studies of microbiome-targeted organic amendment comes from Postma and Schilder [27], who demonstrated in a controlled soil bioassay that several organic amendments enhanced suppressiveness against *R. solani* AG 2-2 IIIB (Tier 2 evidence, not a field trial). This result matters because it ties an ordinary agronomic practice to a measurable disease-suppressive outcome in the target crop itself—a combination that remains rare in this literature. However, the effective treatments were applied at 0.3% *w*/*w*, corresponding to amendment rates that may not be economically scalable in commercial production, and field efficacy was not tested.

The efficacy of organic amendments is highly dependent on amendment quality (carbon-to-nitrogen ratio, maturity, composition), application rate, soil type, and timing, and results from one production region should not be extrapolated to others without local validation. The mechanism was not fully resolved: the primary study [27] quantified a specific taxon (*Lysobacter*) by qPCR but stated that the mode of action could not be determined (Tier 2, sugar-beet controlled), meaning that disease reduction may be moderate and cumulative rather than rapid [69,70], with bio-organic amendments inducing functional shifts in rhizosphere bacterial communities [69] and the duration of amendment treatment influencing disease-suppressive outcomes (Tier 3, cross-crop) [70]. For agronomic recommendations, the practical implication is that organic amendments should be viewed as a foundational soil-health practice that supports—but does not replace—other disease management components. Negative results have also been reported: certain amendments can increase specific diseases, and the variability of organic material composition, the degree of compost maturity, and the C:N ratio all influence whether the net effect is suppressive or permissive. These factors should be evaluated in local trials before recommendation.

Biofumigation can also affect non-target soil organisms and reshape microbial communities. Depending on the amendment, dose, soil, and application timing, these effects may include transient losses of particular microbial functions or diversity but may also enrich taxa associated with disease suppression; however, enrichment of taxa or shifts in community structure are not themselves independent evidence of suppressiveness, which requires functional confirmation (Tier 3, cross-crop) [71,72]. This non-selectivity is a critical agronomic caveat: a biofumigation strategy that suppresses *R. solani* but also depletes antagonistic *Pseudomonas* or *Trichoderma* populations may trade short-term pathogen reduction for the long-term loss of suppressive capacity. The net agronomic effect depends on the balance between pathogen sensitivity and beneficial-microbe resilience, which is soil- and amendment-specific. Sugar-beet-specific field evidence directly quantifying both pathogen suppression and microbiome-side effects of biofumigation remains scarce, and biofumigation recommendations for sugar beet should therefore be made cautiously and ideally supported by local trial data with microbiome monitoring.

## 7. Soil Physicochemical Management and Chemical Input Compatibility

Soil physicochemical properties set the bounds within which the microbiome can suppress disease, and several of them—liming, fertilisation, and irrigation—are adjustable. (Unless otherwise noted, general physicochemical–microbiome relationships in this paragraph are Tier 3, cross-crop/indirect.) pH stands out as the strongest abiotic driver of microbial community structure (Tier 3, cross-crop) [73,74], and neutral to slightly alkaline conditions tend to favour antagonistic genera such as *Pseudomonas* and *Streptomyces*. Liming is an established approach for managing *Aphanomyces* root rot in sugar beet: Swedish studies showed that higher soil Ca levels were associated with reduced disease risk and proposed a threshold of approximately 250 mg of Ca/100 g of soil (Tier 1, sugar-beet field observational) [75], while subsequent trials across 52 soils demonstrated that lime amendments reduced *Aphanomyces* root-rot potential (Tier 1, sugar-beet field) [76]. In Minnesota and North Dakota, field application of sugar-beet factory spent lime has likewise been documented to suppress *Aphanomyces* root rot (Tier 1, sugar-beet field) [77]. The microbiome-mediated component of lime’s suppressive effect, however, remains poorly characterised. Acidification reduces the capacity of soil microbiomes to suppress pathogenic *Fusarium* [78], whereas alleviating soil acidity can enrich potentially beneficial rhizobacteria and enhance soil disease suppressiveness (Tier 3, cross-crop) [79]. Liming can also shift pathogen communities and nutrient availability in unintended ways. Soil type and texture also shape community assembly and suppressiveness [80]. Whether light-textured soils consistently carry lower microbiome-mediated suppressiveness in sugar beet still needs direct field testing. Beyond pH, several other physicochemical factors shape microbiome-mediated suppressiveness. Soil moisture and irrigation management are critical because *Aphanomyces* and *Pythium* are moisture-driven oomycetes whose zoospore dispersal depends on free water; excessive irrigation can increase damping-off pressure, while moderate moisture supports antagonistic microbial activity. Temperature modulates both pathogen virulence and antagonist efficacy: *R. solani* AG 2-2 IIIB is most active under warm conditions (Tier 1–2, sugar-beet) [31], *Pythium* diseases are strongly favoured by high soil moisture, whereas temperature responses are species-specific; *P. ultimum* is generally associated with cooler-to-moderate conditions, while *P. aphanidermatum* is favoured by warmer soils [39]. Soil compaction restricts root growth and alters oxygen availability, favouring anaerobic conditions that can shift microbial communities toward pathogen-friendly states. Organic matter content is a key driver of general suppressiveness, as it sustains microbial biomass and activity. In the arid and semi-arid production environments of Inner Mongolia and Xinjiang, soil salinity and sodicity are dominant drivers of microbial assembly and can constrain the establishment of beneficial rhizosphere communities.

Chemical input management is a critical and often underappreciated component of microbiome-compatible disease management. Some fungicides, particularly triazoles applied repeatedly or at elevated concentrations, can reduce soil microbial biomass and activity and alter microbial functional profiles (Tier 3–4, cross-crop/in vitro) [81,82]. Although azoxystrobin is effective against *R. solani* in sugar beet [7], laboratory soil experiments indicate that it can alter bacterial diversity and community composition and affect soil enzyme activities (Tier 4, laboratory soil microcosm; azoxystrobin efficacy [7] is Tier 1, sugar-beet field) [83]. Its microbiome compatibility should therefore be evaluated at field-relevant application rates. Long-term fertilisation regimes can substantially reshape soil microbial diversity and community composition. In the systems examined, organic inputs or combined organic-mineral fertilisation were associated with distinct microbial communities and, in some cases, greater microbial diversity and ecosystem multifunctionality than mineral-only or unfertilised treatments (Tier 3, cross-crop; no pathogen suppression tested) [84,85]. However, these studies did not directly evaluate pathogen suppression or antagonistic activity.

The integrated fungicide-biocontrol field trial of Kiewnick et al. [22] remains one of the few published studies demonstrating combined chemical-biological efficacy against *Rhizoctonia* in sugar beet under field conditions (Tier 1, sugar-beet field evidence). Fungicides alone reduced disease by 50 to 90%, and the combination of azoxystrobin at 76 g of active ingredient per hectare with a *Bacillus* isolate (MSU-127) gave the best disease reduction and greatest root and sucrose yield increase [22]. Notably, low-rate azoxystrobin combined with the *Bacillus* isolate outperformed a substantially higher rate of azoxystrobin applied alone—dose-reduction evidence that supports this review’s argument. The 50–90% reduction covers two fungicides across a fourfold range of rates, and the trials were artificially inoculated; the bacteria were applied at the four-leaf stage, whereas seed and in-furrow applications are realistic commercial delivery routes. This study supports a narrower, evidence-based principle: targeted, reduced-dose fungicide use should be recommended only in experimentally validated BCA–fungicide combinations. In this review, this recommendation refers specifically to the reduced-dose azoxystrobin–*Bacillus* combination tested by Kiewnick et al. [22], not to indiscriminate dose reduction, which can risk insufficient control and selection of less sensitive populations depending on the active ingredient and pathogen. However, the *Bacillus* species identity was not confirmed, the trial addressed only a single pathogen, and the results are now over two decades old; updated integrated trials against the current pathogen complex are needed.

**Figure 4:**
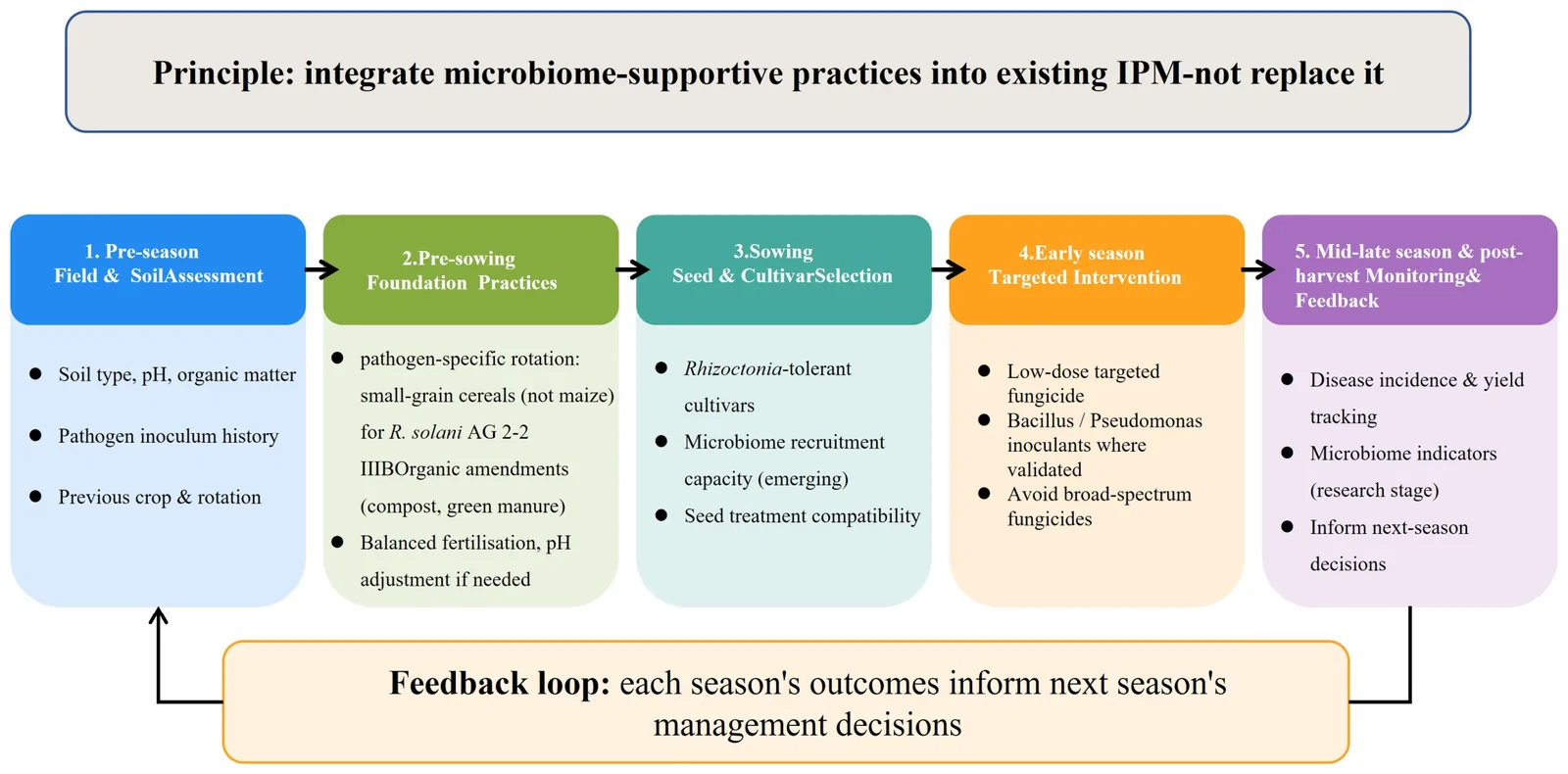
Roadmap for integrating rhizosphere microbiome strategies into sugar-beet soilborne disease management across the growing season. Five sequential stages are shown: field and soil assessment (pre-season), foundation practices including rotation and organic amendments (pre-sowing), seed and cultivar selection (sowing), targeted intervention with reduced-dose fungicides in experimentally validated BCA–fungicide combinations (early season), and monitoring with feedback to inform next-season decisions (mid–late season and postharvest). The guiding principle is to integrate microbiome-supportive practices into existing IPM rather than deploy them as a stand-alone replacement.

For agronomic recommendations, the practical synthesis is that microbiome-oriented soil management should be integrated with cultivar choice, seed treatment, rotation planning, irrigation management, and soil health monitoring rather than promoted as a single replacement for existing disease-control tools. The effects of agricultural practices are especially relevant for sugar beet, where continuous or shortened rotations favour inoculum accumulation and reduce microbial buffering capacity. A seasonal roadmap for integrating these microbiome strategies into existing disease management programmes is presented in Figure 4.

## 8. Microbial Inoculants and Synthetic Communities

Microbial inoculants represent the most direct attempt to deploy rhizosphere microbiome function for disease suppression. (The mechanistic claims in this paragraph are Tier 4, mechanistic inference.) *Bacillus*- and *Pseudomonas*-based products have practical advantages because of their antagonistic metabolites, plant-growth-promoting traits, and compatibility with integrated pest management [86,87,88], with *Bacillus* cyclic lipopeptides exhibiting compound-specific, membrane-targeting antimicrobial activities [88,89,90,91] and combined *Trichoderma*–*Bacillus* applications producing synergistic biocontrol effects [92]. *Bacillus subtilis* and *B. velezensis* produce lipopeptides such as iturins, fengycins, and surfactins, which exhibit activity against diverse fungal phytopathogens, including *Rhizoctonia* and *Fusarium* spp. [87,88,89]. The *Pseudomonas fluorescens* complex contributes 2,4-DAPG production [50,93], phenazine compounds [94,95], pyoverdine siderophores [88], and hydrogen cyanide and volatile organic compound production [96,97]. *Trichoderma* spp. operate through mycoparasitism via chitinases, glucanases, and proteases [98,99]; induced systemic resistance [100]; and competitive exclusion [101]. *Pseudomonas* spp. also trigger induced systemic resistance via jasmonate and ethylene signalling [90,102]. *Streptomyces* spp. produce diverse secondary metabolites with antagonistic activity [103,104].

Despite this mechanistic promise, the field performance of single-strain inoculants in sugar beet has been inconsistent. Recent sugar-beet studies have identified promising *Bacillus* and *Pseudomonas* strains against *R. solani*, primarily under greenhouse or pot conditions (Tier 2, sugar-beet controlled) [105,106], whereas direct field efficacy evidence remains much more limited; Kiewnick et al. [22] provides Tier 1 field efficacy evidence, while Wolfgang et al. [28] contribute cultivar-specific controlled and field observational evidence rather than a comparable field efficacy trial. In greenhouse experiments with sugar beet, Zachow et al. [23] characterised the strain-specific colonisation patterns of five biocontrol agents against *R. solani* AG 2-2 IIIB, including three bacterial strains (*Pseudomonas fluorescens*, *P. trivialis*, and *Serratia plymuthica*) and two fungal strains (*Trichoderma gamsii* and *T. velutinum*); this study focused on colonisation patterns and plant-growth promotion rather than on quantified disease suppression (Tier 2, sugar-beet greenhouse). Field colonisation by *Trichoderma* often falls below laboratory levels, and a pervasive methodological issue is the predominant use of single-strain and single-pathogen experimental designs that fail to capture indigenous microbiome interactions, multi-pathogen co-infection, and field environmental fluctuations. This likely explains the frequent failure of laboratory results to translate to field conditions [107,108] (Tier 3, cross-crop/indirect). Seed treatment and pelleting provide a practical delivery route for biological agents in sugar beet, where commercial seed is commonly supplied as pelleted seed with protective seed treatments. Hymexazol, in particular, is widely used as a commercial-seed treatment against *Aphanomyces* and *Pythium* damping-off during the first few weeks after sowing [109]. Compatibility with pellet formulation and with existing seed-applied chemistry is arguably the single largest determinant of field feasibility for inoculant-based strategies, yet Wolfgang et al. [28] tested seed treatment explicitly and Farhaoui et al. [105] delivered its agent by seed soaking, illustrating that this delivery route is already being explored in the target crop (Tier 2, sugar-beet controlled delivery evidence).

Synthetic communities (SynComs) represent a promising longer-term direction (general SynCom evidence here is Tier 3, cross-crop, unless noted), assembled from known-composition strains according to explicit design principles [110,111], with Cross-kingdom SynComs combining bacterial and fungal members have shown superior suppression to single-kingdom consortia in tomato *Fusarium* wilt [112]. Other cross-crop studies have demonstrated effective bacterial SynComs, including a defined bacterial consortium against *Fusarium* wilt in watermelon [113]. In sugar beet, recent work has instead focused mainly on single bacterial strains or simpler inoculant combinations [114] and machine learning approaches identifying key driver strains (inferred from cross-crop machine-learning association, not validated in sugar beet) for plant protection [115]. By combining strains with complementary functional traits, SynComs can potentially achieve broad-spectrum suppression through functional complementarity, enhanced colonisation stability through niche differentiation, and greater environmental adaptability through functional redundancy [116,117], while beneficial microbes also trigger induced systemic resistance through jasmonate and ethylene signalling pathways [118,119]. As an illustrative hypothetical consortium (not a tested or recommended formulation; Tier 4, untested design), a sugar-beet SynCom might comprise 2,4-DAPG-producing *Pseudomonas* (targeting *R. solani* [120]), antibiotic-producing *B. velezensis* (targeting fungal pathogens), mycoparasitic *Trichoderma* (targeting *R. solani* and *F. oxysporum*), and chitinase-producing *Streptomyces*; however, this proposal requires demonstrated inter-strain compatibility, and the oomycete component (*A. cochlioides* and *Pythium* spp.) is not directly targeted by chitinase-based mechanisms [38] and would require cellulase- or beta-glucanase-producing strains or ISR-inducing taxa for effective suppression. Translating this to the field faces challenges including inter-strain competition, indigenous microbiome exclusion, loss of functional activity in situ, ecological instability of consortia, displacement of introduced strains by native microbiota, high variability of results between field seasons, and a lack of theoretical frameworks for the optimal strain combination design [121]. No sugar-beet field validation of SynComs has been published. The environmental risks of widespread inoculant use also warrant consideration: introduced strains may alter the structure of natural microbial communities, affect uncultivated microbiota, and disrupt existing ecological interactions, with potential long-term consequences that are rarely monitored in field trials (Tier 3–4, cross-crop/mechanistic).

Additional considerations often overlooked in microbiome research include the regulatory framework governing microbial products. Registration requirements for microbial biopesticides differ substantially among jurisdictions: the European Union requires full environmental risk assessment under Regulation (EC) No 1107/2009, the United States Environmental Protection Agency regulates microbial pesticides under FIFRA Section 3, and China has revised its biopesticide registration guidelines to streamline approval for microbial agents [122]. Multi-strain SynCom products face particular regulatory challenges, as most existing frameworks were designed for single-strain formulations and may require each component strain to be individually assessed. Quality control standards for microbial inoculants, including minimum viable cell counts, shelf-life specifications, and contamination thresholds, remain inconsistent across markets. For agronomists, the practical implication is that even when effective SynComs are developed, the path from experimental proof to a commercially available, field-applicable product is long and jurisdiction-dependent. Standardisation of microbiome technologies remains a critical barrier: the reproducibility of inoculant formulations, quality control during production, stability of microbial consortia during storage and transportation, and consistency of viable cell counts all vary across manufacturers and jurisdictions. Without harmonised standards, field-to-field and batch-to-batch variability undermines commercial reliability. Economic aspects also require attention: the cost of production, scaling from laboratory to industrial quantities, economic efficiency relative to existing chemical controls, and the overall readiness of microbiome technologies for practical deployment are rarely analysed in the literature but are decisive for grower adoption. The gap between efficacy under controlled conditions and instability in field trials remains one of the key obstacles to commercialisation of most microbial products: soil type, moisture, temperature, organic matter, native microbiota, and pathogen pressure all shift the outcome (Tier 3, cross-crop evidence on lab-to-field variability) [123,124], and this variability is the central translational problem for inoculant-based strategies.

## 9. Emerging Tools: Omics Diagnostics and Microbiome-Informed Breeding

High-throughput omics and machine learning open two applied routes for sugar-beet disease management: the diagnostic prediction of disease risk and microbiome-informed cultivar selection. (The omics-method statements in this paragraph are Tier 4, methodological/mechanistic.) Root exudate-mediated microbiome assembly is increasingly seen as the mechanism that structures disease-suppressive rhizosphere communities [125,126,127]. Amplicon sequencing (16S rRNA and ITS) is the workhorse technology—being cheap, with mature pipelines—but it carries primer bias, genus-level resolution ceilings, and no functional information [128,129]. Shotgun metagenomics clears those limits, delivering species- and strain-level taxonomic and functional data at once [130]. Mendes et al. [12] used PhyloChip-based community profiling (not shotgun metagenomics) in sugar-beet suppressive soils to link NRPS gene clusters to disease suppression (Tier 2, sugar-beet field-derived soil + greenhouse bioassay). Metatranscriptomics profiles condition-dependent microbial transcriptional activity and therefore provides information that cannot be obtained from DNA-based community profiling alone, and metabolomics gives a functional readout by detecting antagonistic substances, signalling molecules, and metabolic products directly [131,132]. Critical limitations of these methods should be noted: amplicon sequencing suffers from primer bias, genus-level resolution ceilings, platform-dependent biases, and bioinformatic pipeline variability that affects reproducibility between studies; taxonomic annotation depends on database completeness and quality, which is uneven across microbial groups; and shotgun metagenomics, while more powerful, remains cost-prohibitive for routine diagnostic use and faces challenges in strain-level resolution and functional annotation.

Yuan et al. [25] demonstrated that random forest models trained on multi-crop rhizosphere data (banana, cucumber, watermelon, lily, but not sugar beet) can predict *Fusarium* wilt occurrence with 80% field validation accuracy, illustrating how omics data can be translated into practical early-warning tools (Tier 3, cross-crop; Yuan et al. [25] used non-sugar-beet crops), with deep learning approaches further improving prediction accuracy for soilborne fungal diseases from microbiome data [133] and reference-free metagenomic sequencing enabling plant disease detection without prior pathogen identification [134]. However, key challenges limit broader adoption: models trained on one site often fail to generalise across soil types and climates; high-dimensional sparse datasets are prone to overfitting; and statistically identified marker taxa may not be causally linked to disease outcomes [135]. Future work should prioritise simple, validated microbial or metabolic indicators that can be linked to disease risk and management response across diverse field conditions, rather than exhaustive community descriptions. Kusstatscher et al. [136] showed that microbiome-derived indicators can predict sugar-beet disease, illustrating that indicator-based forecasting is already feasible in this crop, though their study addressed postharvest rather than field soilborne disease (Tier 3, sugar-beet postharvest). It is important to distinguish pathogen diagnostics from microbiome-based diagnostics. For sugar beet, pathogen-specific molecular tools are already available for several major soilborne diseases. A quantitative real-time PCR assay for *Aphanomyces cochlioides* has been developed and validated using naturally infested field soils, showing a strong relationship between pathogen DNA abundance and inoculum density and providing a potential tool for identifying fields at high risk of *Aphanomyces* root rot (Tier 2, sugar-beet validated assay) [137]. Likewise, a specific qPCR assay has been developed for *Rhizoctonia solani* AG 2-2 IIIB, enabling quantification of pathogen DNA and supporting estimation of inoculum in soil and infected plant material (Tier 2, sugar-beet validated assay) [138]. Microbiome-derived functional markers would therefore need to provide information beyond pathogen abundance alone—for example, by improving disease-risk prediction across contrasting soil conditions or by integrating intrinsic soil suppressiveness with pathogen load. A comparable benchmark also exists on the host side: the *RsBv1* SNP on chromosome 6 is significantly associated with resistance to *R. solani* AG 2-2 IIIB and has been developed as a practical marker for marker-assisted selection in sugar beet [139]. (Tier 1, direct sugar-beet field/genetic association). Thus, future microbiome-informed diagnostic or breeding approaches should be evaluated against these established pathogen-quantification and host-resistance tools rather than considered in isolation.

Microbiome-informed breeding is a further emerging opportunity. Wolfgang et al. [28] showed that sugar-beet cultivars differing in *Rhizoctonia* tolerance recruit different rhizosphere communities, though cultivar effects on root communities were partial, with substrate being a stronger determinant of root community structure and seed communities the most differentiated compartment, suggesting that host genetics can modulate disease-relevant microbial functions (Tier 2, sugar-beet greenhouse + field observation). Incorporating microbiome recruitment capacity into sugar-beet breeding objectives is highly promising yet little explored [140], though host genetics are increasingly recognised as regulators of the plant microbiome [141], and engineering the crop microbiota through host genetics offers a viable breeding strategy [142] (Tier 3, cross-crop/general breeding evidence). For agronomists, the near-term implication is that cultivar selection can be informed not only by direct disease resistance scores but also by documented differences in microbiome recruitment, although standardised protocols for assessing microbiome recruitment capacity are not yet available. Critically, the same study [28] reported that applying bacterial control agents to *Rhizoctonia*-tolerant cultivars produced no benefits and, in some cases, slightly reduced plant health, interpreted as interference with native root bacteria (Tier 2, sugar-beet controlled evidence). This documented negative genotype-by-inoculant interaction in the target crop is one of the most directly relevant sugar-beet-specific observations illustrating the translational gap, and it directly qualifies the recommendation to apply *Bacillus*/*Pseudomonas* inoculants without reference to cultivar.

## 10. Challenges and Priority Research Directions

Rhizosphere microbiome research has moved fast, but several barriers stand between current knowledge and routine microbiome-based management of sugar-beet disease. Causation is the first. Most studies correlate microbiome composition with disease outcomes; pinning down causality will need controlled experiments—SynCom inoculation, soil transplantation, and gene knockout validation [143]—and disease-suppressive soils are a useful but underused model for that dissection [144]. Scale is the second: short laboratory runs cannot capture multi-year field dynamics, and this lab-to-field gap is among the hardest translational hurdles, because soil type, moisture, temperature, organic matter, native microbiota, and pathogen pressure all shift the outcome [123,124]. Co-infection is the third: the sugar-beet disease complex mixes fungi and oomycetes with fundamentally different types of infection biology, so single-pathogen designs fall short of practical recommendations. A fourth barrier is the microbiome–host genotype interaction—cultivar differences in rhizosphere microbiomes have been reported [28], but the underlying genetic and molecular mechanisms are unclear. Fifth, distinctive sugar-beet root traits are a barrier: its documented organic-acid exudation, fleshy taproot architecture, and the still unresolved contribution of glycine betaine to root exudation and microbial recruitment. Sixth, climate change: warming and extreme precipitation could rewire pathogen–microbiome–plant dynamics, yet sugar-beet-specific evidence is thin, and arid and semi-arid production environments need targeted studies [145,146]. Key research gaps that specifically limit the introduction of microbiome technologies into production include: (1) the lack of multi-site and multi-year field validations of SynComs in the target crop; (2) the absence of standardised protocols for assessing microbiome recruitment capacity in breeding; (3) the uncharacterised microbiome-mediated component of established practices, such as liming; (4) the missing link between microbiome markers and actionable management thresholds; (5) the need for seed-treatment-compatible biological delivery systems specific to pelleted sugar-beet seed; and (6) the economic and regulatory barriers to scaling from experimental proof to commercially available product.

Ordered by how readily they translate to the field, the priority research directions are these. Long-term, multi-site field experiments across the main sugar-beet regions of China, Europe, and North America should track disease incidence, yield, pathogen dynamics, and microbiome composition together under standard management. Multi-pathogen challenge experiments that mirror field co-infection would give realistic efficacy estimates for biocontrol agents and SynComs. The effects of organic amendments and rotation on suppressiveness need validation across sugar-beet soil types and climate zones, with microbiome monitoring to separate generalisable from site-specific responses. Multi-functional SynComs designed for the sugar-beet pathogen complex require multi-site and multi-year field testing. Cost-effective microbial or metabolic indicators, calibrated to local conditions, would support field-level disease risk assessment. And microbiome recruitment capacity should be built into sugar-beet breeding evaluation systems.

## 11. Conclusions

The rhizosphere microbiome is a useful but uneven resource for integrated protection against sugar-beet soilborne disease complexes. The strategy that pays off soonest is not to swap out established controls for microbial tools but to fold microbiome-supportive practices into the disease management programme already in place. Diversified rotations with suitable non-host crops can support general suppressiveness, though direct sugar-beet field evidence for microbiome-mediated rotation effects remains limited (Tier 1 evidence is restricted to pathogen-incidence outcomes without microbiome monitoring; microbiome-level rotation evidence is Tier 3, cross-crop). Organic amendments can support general suppressiveness, while targeted, reduced-dose fungicide use in experimentally validated BCA–fungicide combinations can improve integrated disease control; soil health monitoring can further support microbiome-compatible management. *Bacillus*-, *Pseudomonas*-, *Streptomyces*-, and *Trichoderma*-based interventions still hold promise, yet their worth for sugar beet will turn on validation under multi-pathogen field conditions rather than single-pathogen assays alone, and most current evidence remains at the laboratory or greenhouse tier (Tier 2–4) rather than at the field-ready tier (Tier 1). Omics-based diagnostics, synthetic communities, and microbiome-informed breeding are longer-horizon opportunities, especially if tied to practical indicators of disease risk and management response. Inoculant recommendations should be cultivar-specific, as genotype-by-inoculant interactions can produce null or negative results [28].

For agronomists and crop managers, the implications are concrete. Lead with practices supported by the most direct available sugar-beet evidence—integrated fungicide–biocontrol combinations have direct field support, whereas organic amendments have demonstrated enhancements of *Rhizoctonia* suppressiveness in sugar beet under controlled soil-bioassay conditions and still require field-scale validation—ahead of practices backed only by greenhouse or cross-crop data. Read single-pathogen trial results as not necessarily predictive of performance under co-infection, where pathogen interactions can be antagonistic, synergistic, or neutral. Approach biofumigation and broad-spectrum fungicide use with caution: while biofumigation may enrich taxa associated with disease suppression under some conditions (enrichment is associative, not independent evidence of suppressiveness without functional confirmation) (Section 6), it may also restructure non-target microbial communities in ways that are beneficial, neutral, or detrimental depending on amendment type, dose, soil properties, and timing; local trials with microbiome monitoring are therefore needed to determine the net benefit. Likewise, reduced fungicide rates should be recommended only where specific BCA–fungicide combinations and application regimes have been experimentally validated, rather than through indiscriminate dose reduction.

Join or start multi-site and multi-year field trials with microbiome monitoring, because the sugar-beet-specific evidence base is thin, and region-specific validation is the binding constraint. Such trials should ideally measure pathogen abundance, disease incidence, yield, microbiome composition and function, and key environmental variables simultaneously. Watch cultivar-specific microbiome recruitment as an emerging selection criterion, while accepting that standardised protocols are still being worked out. The translation goal is decision-oriented integrated plant protection: prioritise agronomic practices that are supported by the strongest available sugar-beet evidence, while acknowledging that the evidence base for several practices remains incomplete; validate microbial products in realistic field systems; and build crop-specific tools that account for sugar-beet root biology, pathogen diversity and potential co-infection, host genotype, and local soil conditions.

## Data Availability

No new data were created or analysed in this study. Data sharing is not applicable to this article.

## Supplementary Materials

The following supporting information can be downloaded at: https://www.mdpi.com/article/10.3390/plants15182798/s1, Table S1. Key studies linking rhizosphere microbiome traits to sugar-beet soilborne disease suppression, graded by evidence directness (Tier 1: direct sugar-beet field evidence; Tier 2: controlled-environment sugar-beet evidence; Tier 3: cross-crop or indirect evidence; Tier 4: mechanistic/in vitro evidence only). Evidence tiers are assigned to individual claims, not to publications; a single study may contribute evidence at several tiers. Seasons, locations, and field designs are noted for each field study, so that Tier 1 evidence is not treated as uniform [12,22,23,24,25,27,28,56,63,105,106,114,136]; Table S2. Current microbiome-oriented management practices for soilborne disease control in sugar beet and their major limitations [7,22,23,26,27,29,54,55,56,57,64,65,66,67,68,71,72,73,74,75,76,77,78,79,80,83,84,85,88,89,90,91,105,106,109,110,111,112,113,115]. Table S3. Principal Boolean search strings used for literature identification in Web of Science, Scopus, and PubMed, with thematic sub-strings.
